# Thoracoscopic segmentectomy in children with congenital lung malformation

**DOI:** 10.1038/s41598-023-36700-5

**Published:** 2023-06-14

**Authors:** Seohee Joo, Taeyoung Yun, Chang Hyun Kang, Kwon Joong Na, Samina Park, In Kyu Park, Young Tae Kim

**Affiliations:** Department of Thoracic and Cardiovascular Surgery, Seoul National University Hospital, Seoul National University College of Medicine, 101 Daehak-ro, Jongno-gu, Seoul, 03080 Korea

**Keywords:** Medical research, Paediatric research, Paediatrics, Therapeutics

## Abstract

Congenital lung malformations (CLM) are most commonly treated with a pulmonary lobectomy. However, due to technological advancement, video-assisted thoracoscopic surgery (VATS) segmentectomy is becoming an attractive alternative to VATS lobectomy. This study aimed to evaluate the safety, feasibility, and efficacy of VATS segmentectomy as a lung parenchyma-saving strategy in children with CLM. A retrospective analysis was performed on 85 children, for whom VATS segmentectomy was tried for CLM between January 2010 and July 2020. We compared the surgical outcomes of VATS segmentectomy with the outcomes of 465 patients who underwent VATS lobectomy. Eighty-four patients received VATS segmentectomy and thoracotomy conversion was necessary for one patient for CLM. The mean age was 3.2 ± 2.5 (range 1.2–11.6) years. The mean operative time was 91.4 ± 35.6 (range 40–200) minutes. The median duration of chest tube drainage was 1 (range 1–21) day, and the median length of postoperative hospital stay was 4 (range 3–23) days. There were no postoperative mortality and postoperative complications developed in 7 patients (8.2%), including persistent air leakage in 6 patients (7.1%) and postoperative pneumonia in 1 patient (1.2%). The median follow-up period was 33.5 (interquartile range 31–57) months and there were no patients requiring re-intervention or reoperation during the follow-up period. In the VATS segmentectomy group, the persistent air leakage rate was higher than in the VATS lobectomy group (7.1 vs. 1.1%, *p* = 0.003). Otherwise, postoperative outcomes were comparable between the two groups. VATS segmentectomy in children with CLM is a technically feasible alternative to VATS lobectomy with acceptable early and mid-term outcomes. However, the persistent air-leakage rate was higher in VATS segmentectomy.

## Introduction

Congenital lung malformations (CLM) is a spectrum of diseases including congenital pulmonary airway malformations (CPAMs), intralobar sequestration, extralobar sequestration, bronchogenic cysts, and congenital lobar emphysema (CLE). Congenital cystic pulmonary malformations (CPAM) are rare developmental anomalies, characterized by abnormal overgrowth of terminal bronchioles and alveolar suppression. Pulmonary sequestrations are nonfunctional lung parenchyma, which does not connect to the tracheobronchial tree and have a systemic vascular supply.

Although CLMs are often asymptomatic, the frequency of early detection is increasing with improved prenatal imaging^1^. Untreated lesions can lead to recurrent infection, respiratory distress, compressive syndromes, and malignancy^2^. To avoid these long-term complications, the treatment of choice for CLM is a surgical resection with pulmonary lobectomy most commonly performed^2–4^. However, the optimal extent of resection is controversial with some studies advocating for lung parenchyma-saving resections. Considering that CLM is usually diagnosed in neonates and infants with growth potential, lobectomy can trigger complications associated with a reduced lung volume, especially in those with lesions involving multiple lobes^5^. With the improvements in surgical technology, video-assisted thoracoscopic surgery (VATS) parenchyma-saving resection allowing the preservation of normal and healthy lung parenchyma is becoming an attractive alternative to VATS lobectomy.

The aim of this study was to evaluate the safety, feasibility, and efficacy of VATS segmentectomy as a lung parenchyma-saving strategy in children with CLM.

## Materials and methods

### Patients

A retrospective institutional review of 85 consecutive patients, for whom thoracoscopic segmentectomy was tried for the treatment of congenital pulmonary malformations at Seoul National University Children’s Hospital between January 2010 and July 2020, was performed. Among the 95 patients that received a segmentectomy during the study period, 10 patients who underwent a segmentectomy via a posterolateral muscle-sparing thoracotomy were excluded. In the early period of this study, we performed thoracoscopic segmentectomy only in highly selected patients. Currently, we are performing thoracoscopic segmentectomy in almost all patients. Therefore those patients who underwent open thoracotomy were not included in this study. This study has been performed in accordance with the Declaration of Helsinki and approved by the Institutional Review Board of Seoul National University Hospital (IRB number 2105-183-1222). The need for consent was waived by the Institutional Review Board of Seoul National University Hospital (IRB number 2105-183-1222) because of retrospective nature of the study".

If the patients were born in our hospital, a surgeon can meet the parents and patients within a month after birth. However, for the patients referred from other hospitals, the first clinic visiting day was heterogeneous. All patients underwent a preoperative computed tomography (CT) of the chest to evaluate the extent of resection required. A chest CT scan was checked one year after the birth. To minimize X-ray exposure in children, we have tried to minimize the number of CT scans performed whenever possible. If the situation of the patient was not changed, we did not perform an additional chest CT scan. If any persistent lesions are detected in a chest CT scan, surgical resection was recommended in all patients. If pneumonia developed in a patient, we operated on the lesion as soon as possible.

Thoracoscopic segmentectomy was performed if complete resection could be obtained. For asymptomatic children with CLM undergoing elective surgery, surgical resection was planned at least 12 months after birth. The extent of segmental resection was determined based on preoperative chest CT findings. Complex multi-segmentectomy was performed after 2 years old age if there was not any reason to perform urgent surgery. The indication and timing of surgery were determined in consultation with pediatricians and thoracic surgeons.

Data collected from charts included age at operation, sex, height and weight at operation, prenatal diagnosis, preoperative symptoms, type of resection, operative time, surgical pathology, time of chest tube placement, length of hospital stay from operation to discharge, and length of follow-up. Postoperative complications included persistent air leakage, postoperative pneumonia, hemorrhage, pneumothorax, atelectasis, and mortalities. We compared the results of the 456 patients who received VATS lobectomy in our institution during the study period to those of the patients who underwent VATS segmentectomy for the comparison to VATS lobectomy. Routine post-surgical follow-up was performed biannually if the patient has no problem associated with surgery.

### Anesthesia and operative approach

The administration of anesthesia was carried out with general anesthesia and one-lung ventilation. The patients were positioned in lateral decubitus position and CO_2_ insufflation into the thoracic cavity was not used. The Fogarty balloon catheter (Edwards Lifesciences, Irvine, CA) was used to block the major bronchus during single-lumen endobronchial intubation. Our institute's one-lung ventilation technique was described in detail in a prior study^6^. In a lateral decubitus position via three ports—one 20 mm working port usually at the 6th ICS anterior axillary line, one 3 mm camera port at the 8th ICS mid axillary line using a 3 or 5 mm trocar, and one 3 mm instrumental port at the 7th ICS posterior axillary line using a 3 mm trocar. An endoscopic grasper with a 3-mm diameter was used for a 3-mm port; a 3-mm or 5-mm endoscope was introduced through the camera port; and endoscopic staplers were introduced through the working port. Segmental vessels and bronchial branches were individually dissected and clipped with laparoscopic plastic locking clips (Hemolok™) and then divided using a bipolar diathermy sealing device (LigaSure™). The lung parenchyma was divided using endoscopic staplers (Signia™ Stapling system using EndoGIA™ 45 mm tan or purple reload cartilages). The lung was expanded to check for non-ventilated residual lung parenchyma. A chest tube was introduced in one of the port sites after the lesion was removed. Furthermore, a 4 K endoscope and surgical monitor were used to aid in an accurate and precise operation.

### Considerations to avoid incomplete removal of CLM

The primary concern during segmentectomy in young children is preventing remaining CML lesions. In our institute, we have implemented a number of principles to prevent CLM from being partially resected.

1. Prior to surgery, 3D reconstruction chest CT imaging should be used to assess the precise location and size of the lesion. To determine the proper operation planning, this phase is essential.
2. Until a child is two or three years old, complex segmentectomy should be delayed. If a patient is too small, it is difficult to control small segmental branches and locate the exact parenchyma division plane.
3. All hilar structures should be extensively dissected during the segmentectomy to the level of the lobar bronchus or artery. The hilar attachment should be entirely dissected from the lung parenchyma of the targeted segment. To guarantee complete excision of the target segment, the parenchyma division line should be placed very close to the adjacent segmental bronchus or arteries.
4. Our Institute does not recommend routinely performing follow-up chest CT scans on developing children to repeatedly expose them to x-rays. Nonetheless, the chest CT scan should be evaluated to search for any residual CLM lesion if a patient develops any signs of recurrent respiratory infection.

### Statistical analysis

Continuous variables were expressed in mean ± standard deviation or medians with ranges; Categorical variables were presented in percentages and frequencies. Student t-tests were used for continuous variables and the chi-square test for nominal variables to compare the two groups. Statistical significance was defined as a *P* value of 0.05. Data were analyzed with SPSS software ver. 12.0 (SPSS Inc., Chicago, IL, USA).

## Results

### Baseline characteristics

**Figure 1:**
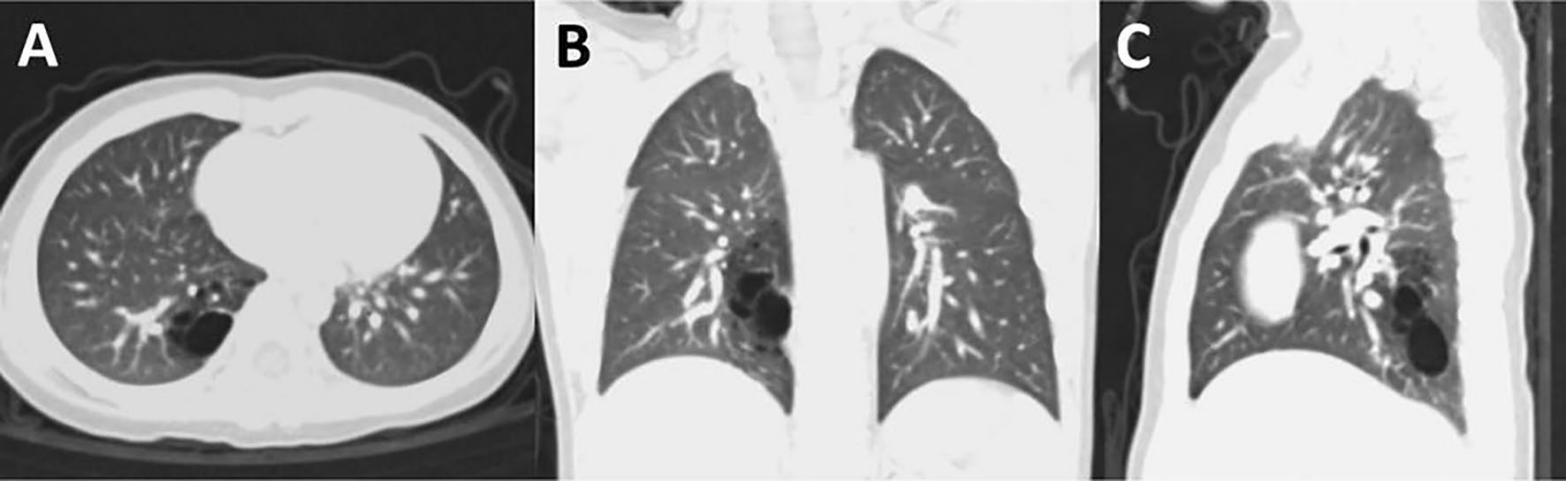
Congenital pulmonary airway malformation limited to the posterior basal segment (S10) of the right lower lobe on a chest computed tomography scan. (**A**) axial view (**B**) coronal view (**C**) sagittal view.

**Figure 2:**
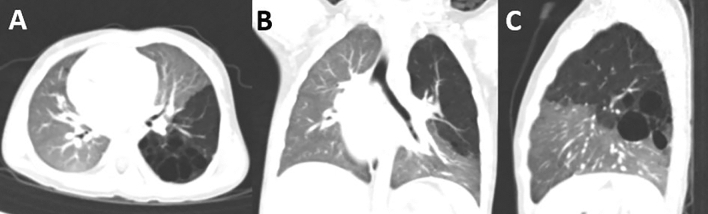
Congenital pulmonary airway malformation involving multiple lobes on a chest computed tomography scan: the apicoposterior segment, anterior segment (S1-3) of the left upper lobe and superior segment (S6) of the left lower lobe. (**A**) axial view (**B**) coronal view (**C**) sagittal view.

Among 85 patients for whom thoracoscopic segmentectomy was tried, 84 patients underwent thoracoscopic segmentectomy for CLM between January 2010 and July 2020. One thoracotomy conversion was necessary and the conversion rate was 1.2%. Of the 85 patients, 46 (54%) were male and 39 (46%) were female. 71 (84%) were diagnosed with CLM prenatally. All patients performed a chest CT prior to surgery. 43 (51%) patients had right-sided lesions (Fig. 1); 42 (49%) had left-sided lesions (Fig. 2). The mean age at operation was 3.2 ± 2.5 (range 1.2–11.6) years. Before the operation, 26 (31%) patients had histories of pneumonia; Among the 26 patients, two (2.4%) showed respiratory distress at the operation (Table 1).

### Intraoperative data

**Figure 3:**
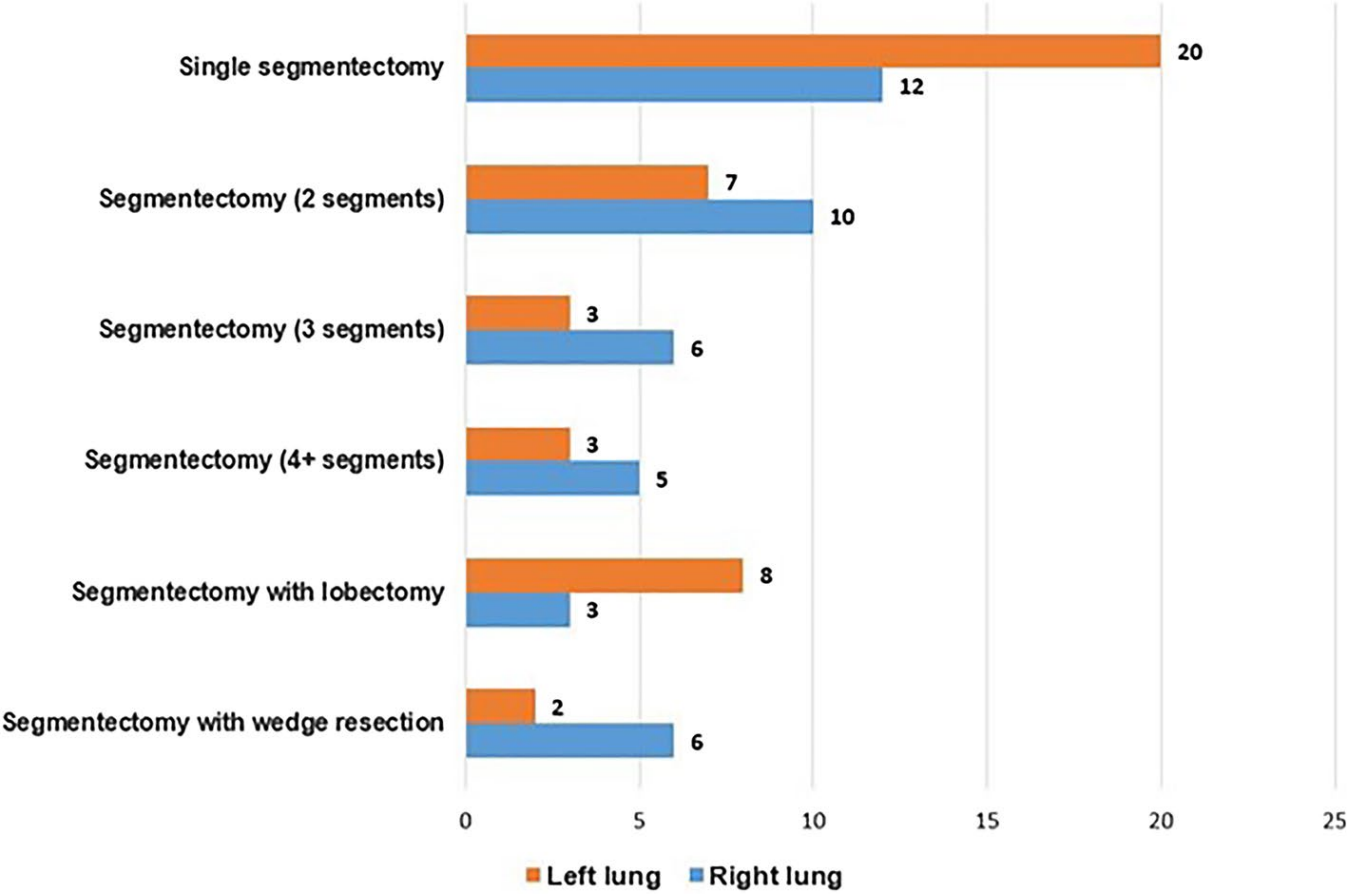
The extent of lung resection and the number of segments resected.

32 (37%) patients underwent isolated segmentectomy for a single segment, 34 (40%) underwent multi-segmentectomy for multiple segments. 19 (22%) patients with lesions in multiple lobes underwent segmentectomy along with a wedge resection and lobectomy as a combined operation; 15 (18%) patients underwent segmentectomy with a wedge resection, 11 (13%) underwent segmentectomy with a lobectomy (Fig. 3) The resected segments are very heterogenous and the locations of segments are listed in Table 2. The mean operative time was 94.4 ± 35.6 (range 40–200) minutes.

**Table 1:** Baseline characteristics of 84 patients with congenital lung malformation.

	N (%)	Mean ± SD	Median	Range
Age (y) at operation		3.2 ± 2.5	2.1	1.2 – 11.6
Gender				
Male	46 (55%)			
Female	39 (45%)			
Height at operation (cm)		94.6 ± 18.1	88.1	76.6 – 159.6
Weight at operation (kg)		15.1 ± 8.5	12.3	8.2 – 60.9
Prenatal Diagnosis	71 (84%)			
Symptomatic	26 (31%)			
History of pneumonia	26 (31%)			
Respiratory Symptoms at operation	2 (2.4%)			
Pathologic diagnosis				
Congenital pulmonary airway malformation	80 (94.1%)			
Type 1	10 (11.8%)			
Type 2	38 (44.7%)			
Type 3	17 (20.0%)			
Type 4	1 (1.2%)			
Mixed type	4 (4.7%)			
Combined with pulmonary sequestration	8 (9.4%)			
Combined with adenocarcinoma	1 (1.2%)			
Pulmonary sequestration	5 (5.9%)			
Congenital lobar emphysema	1 (1.2%)			

### Clinical outcomes

All patients had a chest tube placed intraoperatively. The median duration with a chest tube drainage was 1 (range 1–21) day, and the median length of postoperative hospital stay was 3 (range 2–22) days (Table 3).

**Table 2:** The segments which were resected.

Right	Number (%)	Left	Number (%)
S1	5 (5.9%)	S1 + S2	21 (24.7%)
S2	8 (9.4%)		
S3	5 (5.9%)	S3	13 (15.3%)
S4	0 (0%)	S4	1 (1.2%)
S5	2 (2.4%)	S5	1 (1.2%)
S6	9 (10.6%)	S6	23 (27.1%)
S7	17 (20.0%)	S8	9 (10.6%)
S8	7 (8.2%)		
S9	6 (7.1%)	S9	4 (4.9%)
S10	15 (17.6%)	S10	9 (10.6%)

*Because of multi-segmental resection, the sum of the resected segments exceeds 100%.

### Postoperative complications

There was no postoperative mortality and no patients experienced significant bleeding or required reoperation for any reason. Postoperative complications occurred in 7 (8.2%) patients, which included 6 (7.1%) patients with persistent air leakage, and one (1.2%) patient with postoperative pneumonia. Persistent air leakage was defined as postoperative air leakage for 5 days or longer. The persistent air leakage was resolved without any additional treatment in all patients. The chest tube indwelling time for those patients was 9.1 ± 4.7 days.

We compared these results with those of 456 patients who received VATS lobectomy during the same period at our institution in order to compare the outcomes of VATS segmentectomy and VATS lobectomy (Table 3). All but persistent air leakage were comparable between the two groups in terms of outcomes. The segmentectomy group had a considerably higher rate of air leakage. (7.1% vs. 1.1%, *p* = 0.003).

**Table 3:** Comparison of outcomes and complications according to the extent of surgical resection.

	Segmentectomy (n = 85)	Lobectomy (n = 456)	*p* value
Operative time (minutes)	91.4 ± 87.4	87.4 ± 40.2	0.388
Duration of Chest tube drainage (days)	2.4 ± 3.2	2.2 ± 3.7	0.559
Postoperative length of hospital stay (days)	3.9 ± 3.6	3.7 ± 4.7	0.792
Postoperative mortality	0	0	
Postoperative complication	7 (8.2%)	43 (9.4%)	0.727
Persistent air leakage	6 (7.1%)	5 (1.1%)	0.003
Postoperative pneumonia	1 (1.2%)	2 (0.4%)	0.402
Hemorrhage	0 (0%)	3 (0.7%)	1.000
Pneumothorax	0 (0%)	16 (3.5%)	0.089
Atelectasis	0 (0%)	8 (1.8%)	0.618
Others	0 (0%)	9 (2.0%)	0.367
Follow-up period (months)	42.3 ± 22.3	45.6 ± 27.8	0.288

The patients who underwent combined lobectomy and segmentectomy were compared to those who underwent segmentectomy only or segmentectomy combined wedge resection. The complication rate was 18.2% in the combined lobectomy and segmentectomy group and 6.8% in the segmentectomy group (*p* value = 0.223). The length of hospital stay and chest tube indwelling time was longer in the combined lobectomy and segmentectomy group (3.4 ± 2.6 days vs. 6.9 ± 6.8 days in hospital stay; 2.1 ± 2.2 days vs. 4.8 ± 6.7 days in chest tube indwelling time). However, the statistical significance of the differences was insignificant (*p* = 0.123 and *p* value = 0.211, respectively).

### Follow-up

The median outpatient follow-up duration was 33.5 (interquartile range 31–57) months (Table 3). Follow-up visits were scheduled with a routine plain chest radiography. A routine postoperative chest CT was not performed unless there were respiratory complications requiring an extensive workup. None required readmission or re-operation due to postoperative complications. Four patients had a single episode of respiratory infection during the follow-up period, but CT imaging was not performed since it was thought the infection was not associated with the residual lesion. No patient had recurrent respiratory infections.

To evaluate the long-term lung function after the operation, a pulmonary function test was performed when a patient was more than 6 years old. Pulmonary function test data was available in 262 patients. The FEV1 of each surgical group were 82.9 ± 12.2% in lobectomy (n = 207), 89.8 ± 15.2% in single segmentectomy (n = 23), 86.0 ± 10.7% in double segmentectomy (n = 11), 81.8 ± 10.9% in triple segmentectomy (n = 6), and 78.9 ± 12.7% in quadruple or more segmentectomy (n = 15). The FEV1 value decreased in proportion to the number of segmentectomies. A statistically significant difference compared to lobectomy was identified only in single segmentectomy (*p* = 0.013).

Lung cancer was diagnosed in a patient with CPAM type II. Mucinous adenocarcinoma was found in the right upper lobe. The right upper lobectomy with combined right lower lobe superior segmentectomy was performed. The tumor size was 5 mm and the pathologic stage was pT1aN0M0 and no further treatment was required. The patient is on regular follow-up in our clinic without any evidence of recurrence for two and a half years after the operation.

## Discussion

The procedure of choice for patients with CLM, although controversial, has been a pulmonary lobectomy due to the risk of infection, respiratory distress, mediastinal compression, or malignancy. Many surgeons prefer a lobectomy over a segmentectomy because a segmental resection carries the risk of incomplete removal of the cystic lesions which may result in long-term recurrent infection or malignant transformation. Although the risk for malignant transformation implies that the cystic lesion should be completely removed, the risk of malignant transformation of cystic lesions to malignancy is estimated to be 2–4%. The actual incidence of malignancy is low^7^. Furthermore, a study showed that the incidence of remnant lesions after a segmentectomy was as low as 6%. A segmental resection did not have a higher rate of remnant lesions compared to a lobectomy^8,9^. Moreover, CLM is generally not distributed throughout an entire lobe, but rather, located in a focal part of a lobe. Occasionally lesions are dispersed over multiple lobes. Segmental resections, especially for lesions in multiple lobes, can prevent lobectomies or pneumonectomies and preserve normal healthy lung parenchyma.

Although a lobectomy may sacrifice excessive amounts of the healthy lung parenchyma, this is generally considered to be well tolerated by children since pediatric patients with growth potential are capable of lung volume expansion through lung parenchymal growth^10^. Despite the ability for compensatory lung tissue growth and remodeling in children after lung resection, a study found a significant reduction in the forced expiratory volume in 1 s and the maximal mid expiratory flow rate as well as a reduction in elastic recoiling after a lobectomy^11^. Thus, a comparable rate of complete resection, a low rate of malignant transformation, and a reduced lung function following a lobectomy suggest that a segmentectomy may be a more thoughtful option in completely removing the cystic lesion without excising excessive normal lung parenchyma^12^. In this study, it was determined that the extent of lung function loss was directly proportional to the number of segmentectomies performed. Notably, single segmentectomy demonstrated a significantly higher FEV1 value compared to lobectomy. This discovery highlights the efficacy of preserving pulmonary function through the utilization of segmentectomy.

With the increased use of prenatal ultrasound and improved imaging qualities, the incidence of early detection of CLM in utero is rising. Once diagnosed, early surgical resection is planned for even small asymptomatic patients. However, surgical timing for elective operations for patients with CLM remains controversial. Many authors agree that elective operations before the development of respiratory symptoms are associated with lower incidences of postoperative complications^1,10,13–16^. Nonetheless, surgery immediately after birth in asymptomatic prenatally diagnosed patients is not ideal due to technical problems such as small intercostal spaces, poor tolerance of one-lung ventilation, and limited visualization^17^. Not only is surgical timing crucial, but adequate experience is also key to performing safe and technically demanding thoracoscopic surgeries in children^18^. In this study, elective thoracoscopic segmental resections were performed in asymptomatic patients older than 12 months of age in order to increase the probability of complete resection of the cystic lesion under a safer condition with fewer postoperative complications.

Surgical resection of the cystic lesions can be performed thoracoscopically or by a thoracotomy. However, a thoracoscopic surgical resection should be considered as the initial option due to higher surgical accuracy in the complete removal of the lesion. Thoracoscopic resections are associated with a shorter hospital stay, improved pain control, and a potential for decreased risk of postoperative complications^12,19–21^. Moreover, thoracoscopic surgeries have the advantage of avoiding musculoskeletal morbidity associated with a thoracotomy such as winged scapula, scoliosis, and thoracic wall asymmetry resulting from paralysis and atrophy of the latissimus dorsi and serratus anterior muscles^6,22^.

Persistent air leakage after VATS segmentectomy was the most common complication. 7.1% of the patients in this study experienced continuous air leakage lasting more than 5 days. In previous studies, complications associated with air leakage have been reported ranging from 0.9 to 20%^8,23^. A study emphasized the learning curve effect, which can be a major concern for the improvement of surgical outcomes^23^. However, the authors also emphasized the learning curve can be affected by multiple factors and cautious interpretation is required.

The rate of persistent air leakage is significantly higher in the VATS segmentectomy group in this study when compared to VATS lobectomy. In a prior study for a randomized controlled trial of lung cancer, the increased air leakage rate in segmentectomy was documented^24^. According to the study, segmentectomy and lobectomy patients experienced persistent air leakage rates of 6.5 and 3.8%, respectively. Large lung parenchyma should be transected in the segmentectomy group, therefore there is a higher risk of postoperative air leakage than with lobectomy.

Preoperative diagnosis and disease status can influence the surgical outcome. The most crucial factor in avoiding unnecessary surgery is an accurate diagnosis. Pneumonia in acute exacerbation is not a good surgical indication. The state of the lung parenchyma looks worse by acute phase pneumonia. As a result, if surgery is decided upon too early, unnecessary surgery or excessive resection result. After the inflammation has been completely controlled, the lung has to be re-evaluated whether it is caused by CLM or not and how much lung parenchyma should be resected. When considering segmentectomy, we need to pay attention to a number of distinctive aspects of pulmonary sequestration. Pulmonary sequestration is occasionally associated with agenesis of the basal pulmonary artery. In this case, a lobectomy should be carried out. Those who have a high pressure of systemic blood flow result in secondary vascular change, which cannot be reversed even after the division of the feeding vessel. In this case, a lobectomy should be seriously considered. However, it is uncommon in children.

The limitation of this study was a relatively small sample size. Although this study contains a relatively larger sample of patients who have undergone thoracoscopic segmentectomy when compared to other reports, the study is still limited by a small sample size. This study also lacks long-term follow-up data for thoracoscopic segmental resection. Further studies discussing the long-term outcome are necessary. Moreover, this study was designed as a retrospective study reviewing information on those who underwent VATS segmentectomy. In this study, we delayed operations when complex segmentectomy was required. Delaying the operation can lessen the difficulty of the complex operation and lower the open conversion rate. However, delaying can also have the potential to increase the risk of pneumonia while waiting for the operation. Proper surgical timing is necessary considering surgical difficulty and the risk of pneumonia.

In conclusion, VATS segmentectomy in children with CLM is a technically feasible alternative to VATS lobectomy in preserving lung parenchyma. Modern surgical technology has aided in producing acceptable early and mid-term outcomes. Although the rate of persistent air leakage was higher in segmentectomy, it did not affect mortality or other major morbidity. Therefore VATS segmentectomy can be accepted as an alternative procedure in the future. Further research on VATS segmentectomy concerning residual lesions and long-term clinical outcomes is needed.

## Supplementary Information

Supplementary Information.

## Data Availability

All data generated or analyzed during this study are included in this published article and its Supplementary Information files.

## References

[1] Calvert JK, Lakhoo K (2007). Antenatally suspected congenital cystic adenomatoid malformation of the lung: Postnatal investigation and timing of surgery. J. Pediatr. Surg..

[2] Lakhoo K (2009). Management of congenital cystic adenomatous malformations of the lung. Arch. Dis. Child. Fetal Neonatal Ed..

[3] Laberge JM, Puligandla P, Flageole H (2005). Asymptomatic congenital lung malformations. Semin. Pediatr. Surg..

[4] Muller CO, Berrebi D, Kheniche A, Bonnard A (2012). Is radical lobectomy required in congenital cystic adenomatoid malformation?. J. Pediatr. Surg..

[5] Kim HK, Choi YS, Kim K, Shim YM, Ku GW, Ahn KM, Lee SI, Kim J (2008). Treatment of congenital cystic adenomatoid malformation: Should lobectomy always be performed?. Ann. Thorac. Surg..

[6] Seong YW, Kang CH, Kim JT, Moon HJ, Park IK, Kim YT (2013). Video-assisted thoracoscopic lobectomy in children: Safety, efficacy, and risk factors for conversion to thoracotomy. Ann. Thorac. Surg..

[7] Hartman GE, Shochat SJ (1983). Primary pulmonary neoplasms of childhood: A review. Ann. Thorac. Surg..

[8] Johnson SM, Grace N, Edwards MJ, Woo R, Puapong D (2011). Thoracoscopic segmentectomy for treatment of congenital lung malformations. J. Pediatr. Surg..

[9] Lee S, Kim DH, Lee SK (2017). Efficacy of segmental resection in patients with prenatally diagnosed congenital lung malformations. Interact. Cardiovasc. Thorac. Surg..

[10] Shanmugam G, MacArthur K, Pollock JC (2005). Congenital lung malformations-antenatal and postnatal evaluation and management. Eur. J. Cardiothorac. Surg..

[11] McBride JT, Wohl ME, Strieder DJ, Jackson AC, Morton JR, Zwerdling RG, Griscom NT, Treves S, Williams AJ, Schuster S (1980). Lung growth and airway function after lobectomy in infancy for congenital lobar emphysema. J. Clin. Invest..

[12] Fascetti-Leon F, Gobbi D, Pavia SV, Aquino A, Ruggeri G, Gregori G, Lima M (2013). Sparing-lung surgery for the treatment of congenital lung malformations. J. Pediatr. Surg..

[13] Davenport M, Warne SA, Cacciaguerra S, Patel S, Greenough A, Nicolaides K (2004). Current outcome of antenally diagnosed cystic lung disease. J. Pediatr. Surg..

[14] Aziz D, Langer JC, Tuuha SE, Ryan G, Ein SH, Kim PC (2004). Perinatally diagnosed asymptomatic congenital cystic adenomatoid malformation: To resect or not?. J. Pediatr. Surg..

[15] Kim YT, Kim JS, Park JD, Kang CH, Sung SW, Kim JH (2005). Treatment of congenital cystic adenomatoid malformationdoes resection in the early postnatal period increase surgical risk?. Eur. J. Cardiothorac. Surg..

[16] Wi JH, Lee YH, Han IY, Yoon YC, Hwang YH, Cho KH (2008). Surgical treatment of congenital cystic lung disease. Korean J. Thorac. Cardiovasc. Surg..

[17] Sundararajan L, Parikh DH (2007). Evolving experience with video-assisted thoracic surgery in congenital lung lesions in a British pediatric center. J. Pediatr. Surg..

[18] Park S, Kim ER, Hwang Y, Lee HJ, Park IK, Kim YT, Kang CH (2017). Serial improvement of quality metrics in pediatric thoracoscopic lobectomy for congenital lung malformation: An analysis of learning curve. Surg. Endosc..

[19] Albanese CT, Rothenberg SS (2007). Experience with 144 consecutive pediatric thoracoscopic lobectomies. J. Laparoendosc. Adv. Surg. Tech. Part A.

[20] Vu LT, Farmer DL, Nobuhara KK, Miniati D, Lee H (2008). Thoracoscopic versus open resection for congenital cystic adenomatoid malformations of the lung. J. Pediatr. Surg..

[21] Polites SF, Habermann EB, Zarroug AE, Thomsen KE, Potter DD (2016). Thoracoscopic Vs open resection of congenital cystic lung disease- utilization and outcomes in 1120 children in the United States. J Pediatr. Surg..

[22] Bagrodia N, Cassel S, Liao J, Pitcher G, Shilyansky J (2014). Segmental resection for the treatment of congenital pulmonary malformations. J. Pediatr. Surg..

[23] He T, Sun X, Liu C, Yuan M, Yang G, Cheng K, Dai S, Xu C (2023). Learning curve for total thoracoscopic segmentectomy in treating pediatric patients with congenital lung malformation. Surg. Endosc..

[24] Suzuki K, Saji H, Aokage K, Watanabe S, Okada M, Mizusawa J, Nakajima R, Tsuboi M, Nakamura S, Nakamura K, Mitsudomi T, Asamura H (2019). Comparison of pulmonary segmentectomy and lobectomy: Safety results of a randomized trial. J. Thorac. Cardiovasc. Surg..

